# The composition of single-donor apheresis platelet concentrates is influenced by the age of the donor

**DOI:** 10.1038/s41598-025-97916-1

**Published:** 2025-04-18

**Authors:** Anne-Claire Duchez, Charles-Antoine Arthaud, Marie-Ange Eyraud, Amélie Prier, Marco Heestermans, Hind Hamzeh-Cognasse, Fabrice Cognasse

**Affiliations:** 1 Etablissement Français du Sang Auvergne-Rhône-Alpes, Saint-Étienne, France; 2 INSERM, Université Jean Monnet, Mines Saint-Étienne, U 1059 SAINBIOSE, F- 42023 Saint-Etienne, France

**Keywords:** Predictive markers, Adverse effects

## Abstract

The aging population often faces health issues that sometimes necessitate transfusions. Transfusion services are increasingly concerned about the rising number of transfusions and the aging donor population, as both factors are crucial in maintaining the quality of blood donations. In this context, our study aims to measure the bioactive molecule cytokine levels in single donor apheresis platelet concentrates (SDA-PC) based on the donor’s age and to determine whether these cytokines, in conjunction with the donor age, could contribute to transfusion adverse reactions (AR). Our findings indicate that well-known platelet molecules such as sCD62P, as well as IL-13, ADAMTS13, MIP-1α, NGAL, MCP-3, HSAA, GDF-15, CX3CL1, and MDC, were present in SDA-PC. Levels of MIP-1α, GDF-15, and sCD62P increased with donor aging, whereas levels of MDC decreased. In conclusion, most of the cytokine levels detected were elevated in cases of AR and with increasing donor age. Notably, GDF-15 was the only cytokine that showed a positive correlation with age in the context of AR.

**Supplementary Information:**

The online version contains supplementary material available at 10.1038/s41598-025-97916-1.

## Introduction

Blood is the most complex fluid in the human body. Its composition is highly diverse, containing various cells, nutrients, and active biomolecules such as cytokines, chemokines, and proteases. Cytokines are a broad family of small, secreted proteins that function through receptors to regulate immune activities. Chemokines, a subgroup of cytokines, act as chemoattractants for leukocytes.

The composition of blood, in terms of cytokines and chemokines, is influenced by physiological and pathological conditions and among them both illness^1,2^and the aging process^3–5^. The global population is aging, with the World Health Organization estimating that the proportion of individuals over 60 years old will almost double from 12 to 22% between 2015 and 2050. The prevalence of certain pathologies such as cardiovascular disease, neurological disorders, and cancer increases with age. Concurrently, the demand for blood transfusions is also rising. For instance, WHO data indicate that in high-income countries, approximately 76% of all transfusions are administered to patients over 60 years old^6^. According to the 2021 Global Status Report on Blood Safety and Availability from WHO, in Europe, the number of blood donations and transfusion has fallen significantly during this period^7^. This is explained in particular by patient blood management policies and improved inventory management, which helps avoid product expiration.

In light of an aging population and rising transfusion demands, our study aimed to analyze the composition of single donor apheresis platelet concentrates (SDA-PC) with respect to donor age, specifically examining cytokines and other biomolecules. The goal of this research was to optimize platelet concentrate selection based on donor age, thereby informing targeted advertising campaigns to attract future potential donors. Over the course of three years, our group established a prospective cohort of nearly 9,000 blood donors, utilizing various processes for Platelet Concentrate (PC) collection, including Single Donor Apheresis (SDA). Several analyses were performed to assess bioactive molecule levels. In previous studies, we reported that storage time and adverse reactions could be linked to bioactive molecule levels in different PCs^8,9^. The increased expression of sCD40L and sCD62P, primarily produced by platelets, in SDA-PC during storage^10^. However, the presence of cytokines such as IL-1, IL-6, IL-8, RANTES, CD154, TGFβ, INFγ, and VEGF in SDA-PC has been observed, with significant increases in IL-8 and TGFβ during storage^11–14^. Additionally, other cytokines like IL-13 and MIP-1α have been identified in a small group of donors^15^. However, no studies have investigated the donor’s age in relation to bioactive molecule levels and adverse reactions following a PC transfusion. Here, we measured platelet biomolecules (sCD62P) and leukocyte cytokines (MDC, MCP-3, MIP-1α, NGAL, GDF-15, IL-13, CX3 CL1, and ADAMTS13) which were correlated with donor age and the occurrence of adverse reactions following SDA-PC transfusion. We observed several modulations of cytokine levels with donor age, which are reflected in the composition of SDA-PC. Notably, the level of GDF-15 appears to be linked to adverse reactions in combination with the donor’s age.

## Materiel and methods

### Ethic statement

All research was performed in accordance with relevant guidelines/regulations, and informed consent was obtained from all participants in this study. This research had been performed in accordance with the Declaration of Helsinki. More precisely, Single Donor Apheresis - Platelet Concentrates (SDA-PC) were obtained from “Etablissement Français du Sang (EFS) Auvergne-Rhone-Alpes” with 9,206 volunteers recruited between March 2013 and February 2016 giving their informed consent. All the methods & data in the study was approved by EFS‘s institutional review board for ethics (DC-2019-3803 & AC-2020-3959)^16^. The residual SDA-PCs transfused were collected. Only 3,569PCs were sampled. Seventy-nine Adverse Reactions (AR) were reported in SDA-PC upon transfusion.

More information on the blood donor’s characteristics, the repartition of the sample collection (age of donors, sex with the exact number of samples) are available in Table 1. However, we did not have access to clinical data regarding patient histories who receive the transfusion (disease and comorbidity, number of blood product transfusion, especially platelet concentrate, the time of hospitalization, the goal of the transfusion (prophylactic or therapeutic or both)). The adverse reactions reported in our study were associated with the transfusion of SDA-PC, occurring during the transfusion and lasting for several hours afterward.

**Table 1:** Platelet concentrate with or without adverse reaction with donor’s age.

F/M	No AR						AR					
	1267 F			2302 M			27 F			51 M		
Age (year)	[18–29]	[30–59]	[60–70]	[18–29]	[30–59]	[60–70]	[18–29]	[30–59]	[60–70]	[18–29]	[30–59]	[60–70]
n	384	748	135	194	1655	453	8	16	3	5	40	6

### Sample preparation

SDA-PCs were collected as described above^15,17^. Briefly, blood was collected on ACD-A using Trima, a continuous-flow cell separator (Gambro BCT, Lakewood, CO, USA). The SDA-PCs was automatically resuspended in 35% autologous donor plasma and 65% platelet additive solution (PAS-D, Intersol, Fenwal, La Châtre, France; or PAS-E, SSP+, MacoPharma, Mouveaux, France) and stored at 22 ± 2 °C with gentle rotation and shaking (60 rpm) for a maximum of 5 days (after collection was completed) before being issued for transfusion.

The leftovers of transfused PCs (stocked from 0 to 5 days, at the time of the study) were collected. To remove platelets, the leftover PC was centrifuged (402 × g; 10 min), to remove platelets, after which the supernatants aliquoted and frozen at − 80˚C until further use for ELISA analysis.

### Platelet rich plasma stimulation

Citrated blood samples were received and spun at 280 g, 10 min to prepare the Platelet Rich Plasma (PRP). PRPs were stimulated with TRAP (50 µg/ml) during 30 min at 37 °C. Then, residual platelets were discard after centrifugation at 402 g for 10 min at room temperature. Supernatants were analysed by ELISA for IL-13, MIP1α and sCD62P.

### Multiplex-ELISA

To detect and quantify the level of CX3 CL1, MDC, MCP-3, we used HCYTOMAG-60 K-04 from Merck Milipore. To detect and quantify the level of MIP1a and IL13, we used HCYTOMAG-60 K-02 from Merck Milipore. To detect and quantify GDF-15, NGAL, SAA and ADAMTS13, we used HCVD2MAG-67 K-05 from Merck Milipore. All Merck Millipore multiplex ELISAs are based on Luminex technology.

### Statistical analysis

Multiple comparisons were performed Kruskal wallis and 2-ways ANOVA test. In case of paired unparametric data, the statistical test used was Wicoxon test. P-values of 0.05 and lower are considered statistically significant (* *p* < 0.05, ** *p* < 0.01, ***p < 0.001 and **** *p* < 0.0001). Statistical analysis and Spearman correlation was carried out using GraphPad version 6 (GraphPad Software, La Jolla California USA).

### Biorender

The cartoon at Fig. 4 was made via Biorender, Agreement numbers UB273LEXCQ.

## Results

### Modulation of bioactive molecule levels in circulation through aging

**Fig. 1:**
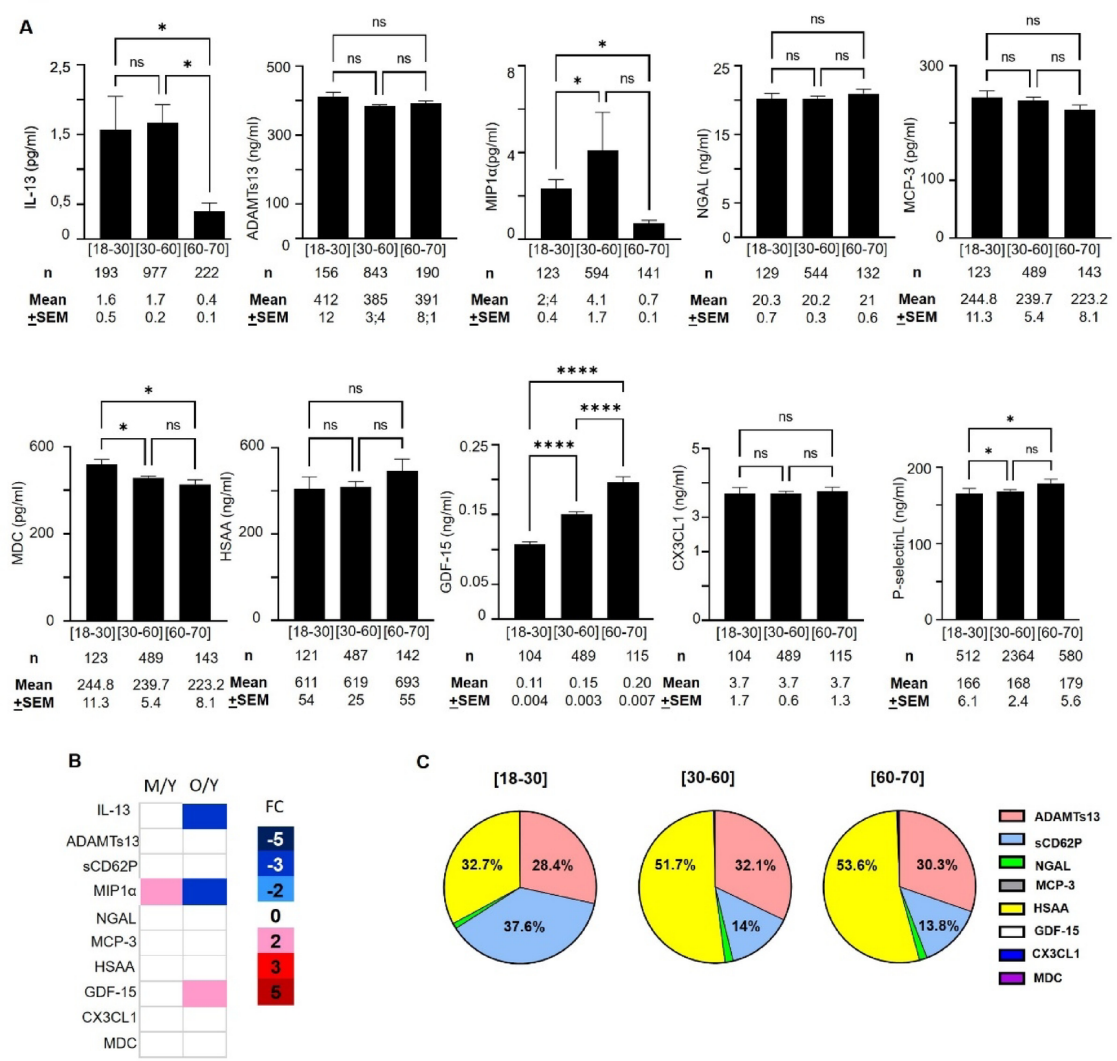
Evaluation of cytokines in Single Donor Apheresis based on donor’s age. (**A**) Graph bars representing the concentration of cytokine in SDA-PC along the donor’s age. Kruskall Wallis test with FDR, * *p* < 0.05;***p < 0.001; ****p < 0,0001. (**B**) Heat Map representing the fold change of the mean between M ([30–59]) and Y ([18–29]) or O ([60–70]). (**C**) Pie chart representing the distribution of the cytokines evaluated in SDA-PC.

When comparing the extreme age groups of donors (18–29 years vs. 60–70 years), we observed modulation in bioactive molecule levels. Specifically, levels of GDF-15 and sCD62P were higher in the older age group, whereas levels of IL-13, MIP-1α, and MDC decreased (Fig. 1A). This observation was supported by fold change calculations between elderly and younger donors, with a fold change (FC) of 2 for GDF-15 and − 4 for IL-13 and MIP-1α (Fig. 1B). Additionally, IL-13 levels decreased, while GDF-15 levels increased between the age groups of 30-59 years and elderly donors (Fig. 1A).

MIP-1α and sCD62P levels increased, whereas MDC levels decreased, when comparing the youngest donors to those aged 30–59 years (Fig. 1A). The MIP-1α level showed a twofold increase between donors aged 30–59 years and those aged 18–29 years (Fig. 1B). The majority of these biomolecules are not synthesized by platelets. However, surprisingly, MIP-1α and sCD62P are released following stimulation of platelet-rich plasma (PRP) with TRAP (Supplemental Fig. 1), in contrast to IL-13. Furthermore, the proportion of this bioactive molecule appeared to increase with age, as observed for HSAA (18–29 years: 32.7%; 30–59 years: 51.7%; 60–70 years: 53.6%), with a similar trend noted for ADAMTs13 (Fig. 1C). However, we observed a decrease in the proportion of sCD62P across the age groups (Fig. 1C).

We subsequently investigated whether donor age or storage duration, influences the bioactive levels, as previously demonstrated^8,9,17^. Within a storage period of 1–3 days, the MIP-1α level significantly increased between the donor age groups of 18–29 and 30–59 years (Supplemental Fig. 2 A). Additionally, during storage periods of either 1–3 days or 3–5 days, GDF-15 levels were significantly higher in donors aged 18–29 compared to those aged 60–70, and in donors aged 30–59 compared to those aged 60–70 (Supplemental Fig. 2 A). The proportion of the various bioactive molecules remained stable across both storage time and donor age groups (Supplemental Fig. 2B).

We then explored whether the bioactive molecule levels in SDA-PC were linked to adverse reactions in relation to the donor’s age. This analysis aims to determine if specific bioactive molecule profiles combined with donor age can predict or explain the occurrence of transfusion-related adverse reactions. Overall, these findings underscore the importance of considering donor age in the evaluation of bioactive molecule profiles in SDA-PC, which could potentially influence the incidence of transfusion-related adverse reactions.

### Modulation of bioactive molecule levels through age and their involvement in adverse reactions following transfusion

Adverse reactions (AR) appears during or after transfusion. Their symptoms included a range of manifestations, such as chills, distress, nausea, fever, rash, edema, and hypertension. First, we investigated whether the age of the donor could serve as a marker for predicting potential adverse reactions. We observed a similar proportion of adverse reactions across different age groups (Supplemental Fig. 3).

**Fig. 2:**
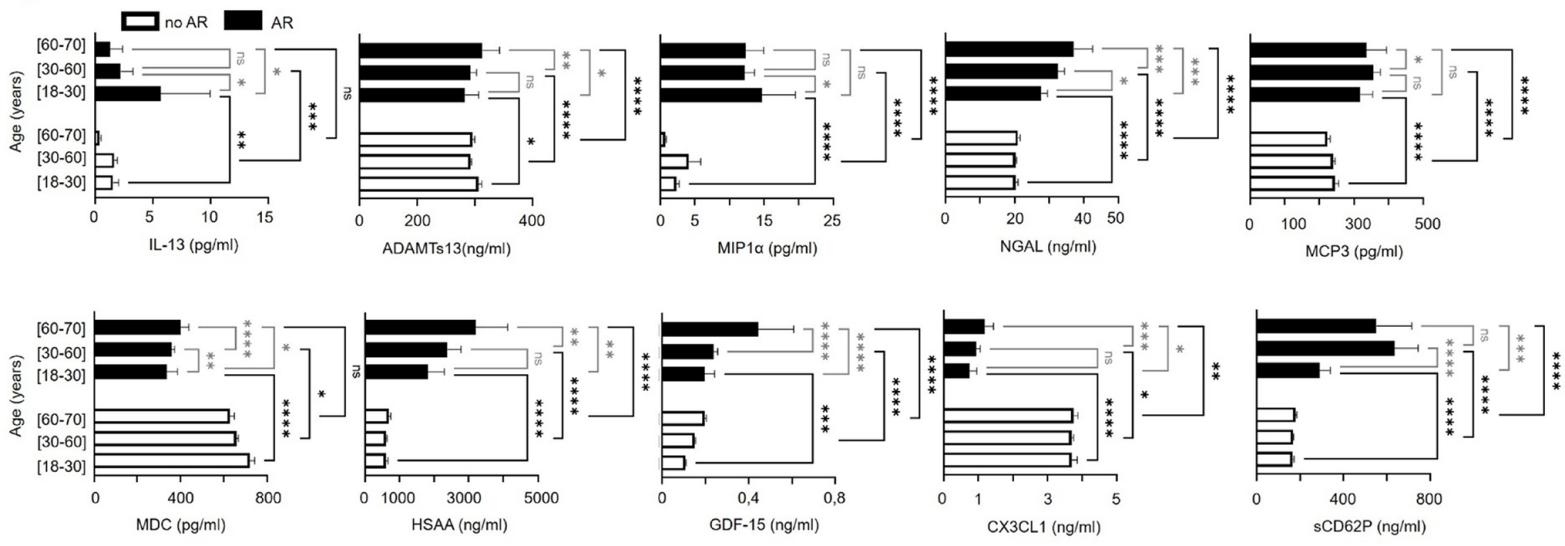
Comparison of cytokines level in Single Donor Apheresis with/out occurrence of AR and based on donor’s age. Graph bars representing the concentration of cytokine in SDA-PC along the donor’s age. 2-ways ANOVA test, * *p* < 0.05; **p < 0;01; *** *p* < 0,001;****p < 0;0001.

Interestingly, within our bioactive molecule panel, ADAMTs13, NGAL, MDC, HSAA, GDF-15, CX3 CL1 and sCD62P levels are increasing with donor’s age in SDA-PC who induced AR after transfusion (Fig. 2). In contrast, IL-13 and MIP1α levels decreased with donor’s age in SDA-PC, which induced AR in the recipient (Fig. 2). Furthermore, when comparing younger donors while ARs concern recipients, we observed significant increases in the levels of IL-13, MIP-1α, NGAL, MCP-3, HSAA, GDF-15, and sCD62P (Fig. 2). Conversely, levels of ADAMTS13, MDC, and CX3 CL1 levels were decreased in younger donors experiencing AR compared to those without AR (Fig. 2). The same pattern of bioactive molecule level changes—both increases and decreases—was observed in the 30–59 year age group when comparing donors with no AR to those with AR (Fig. 2). In elderly donors, bioactive molecule levels such as ADAMTS13, MIP-1α, NGAL, MCP-3, HSAA, GDF-15, and sCD62P increased, while CX3 CL1 levels decreased in the presence of AR (Fig. 2).

**Fig. 3:**
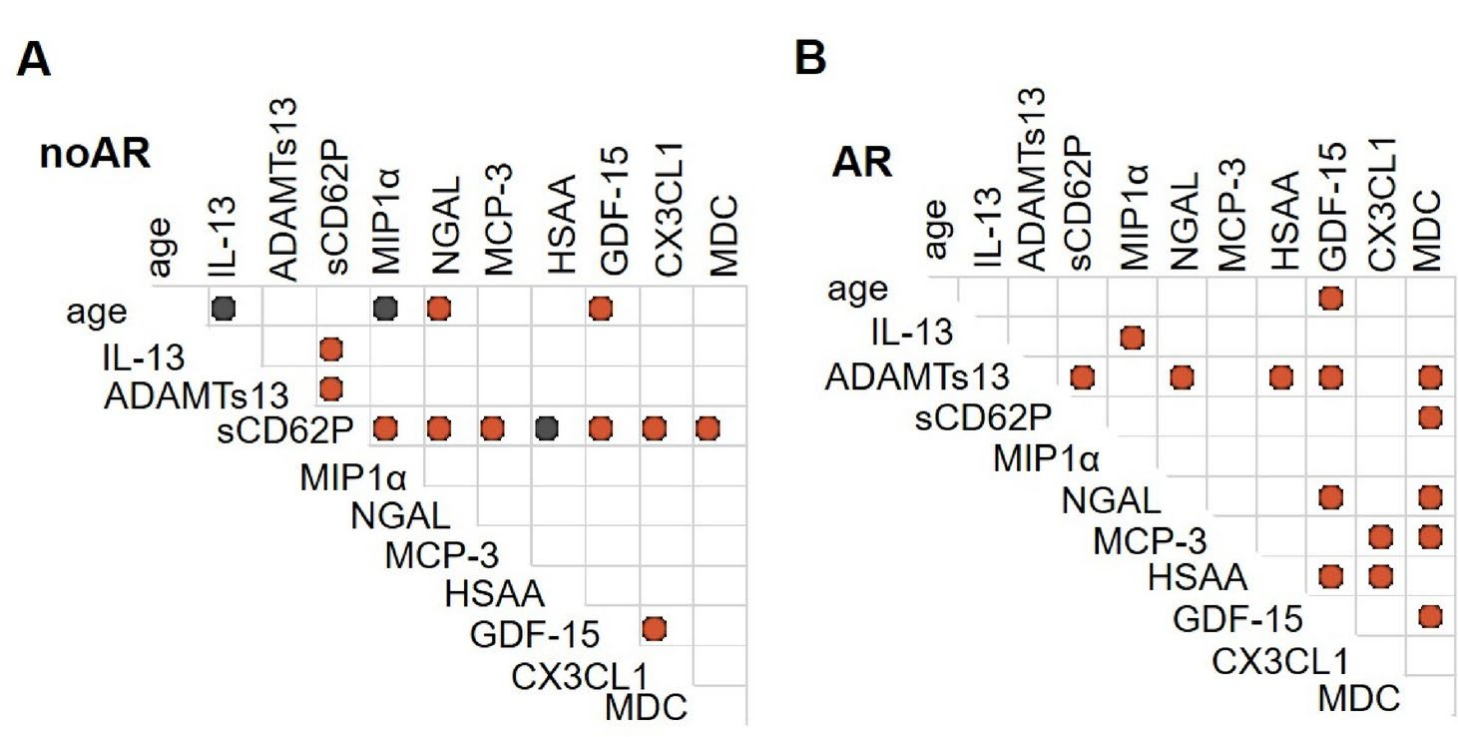
Correlation between cytokine, age and occurrence of AR in SDA-PC. (**A**, **B**) Correlation matrix of cytokine level & age in no AR SDA-PC (**A**), in AR SDA-PC (**B**) Red dots correspond to significant positive correlation between 2 factors, blue dot correspond to significant negative correlation between 2 factors. Matrix of spearman correlation, *p* < 0.05 are considered significant.

In the context of transfusion, we examined whether donor age correlated with bioactive molecule levels. We found that donor age significantly correlated negatively with IL-13, and MIP-1α levels, whereas it significantly correlated positively with NGAL and GDF-15 levels (Fig. 3A). However, a modest correlation was observed between donor age and GDF-15 levels, with a higher correlation coefficient of *r* = 0.52 and a p-value of 3.8 × 10⁻⁵⁰. In cases of adverse reactions, donor age showed a weak positive correlation with GDF-15 levels (*r* = 0.23; *p* = 0.038) (Fig. 3B). Additionally, various bioactive molecule levels displayed either positive or negative correlations with each other, both in the absence of adverse reactions (non-AR) and in the presence of AR (Fig. 3).

## Discussion

### Aging process and bioactive molecule modulation in blood donors

**Fig. 4:**
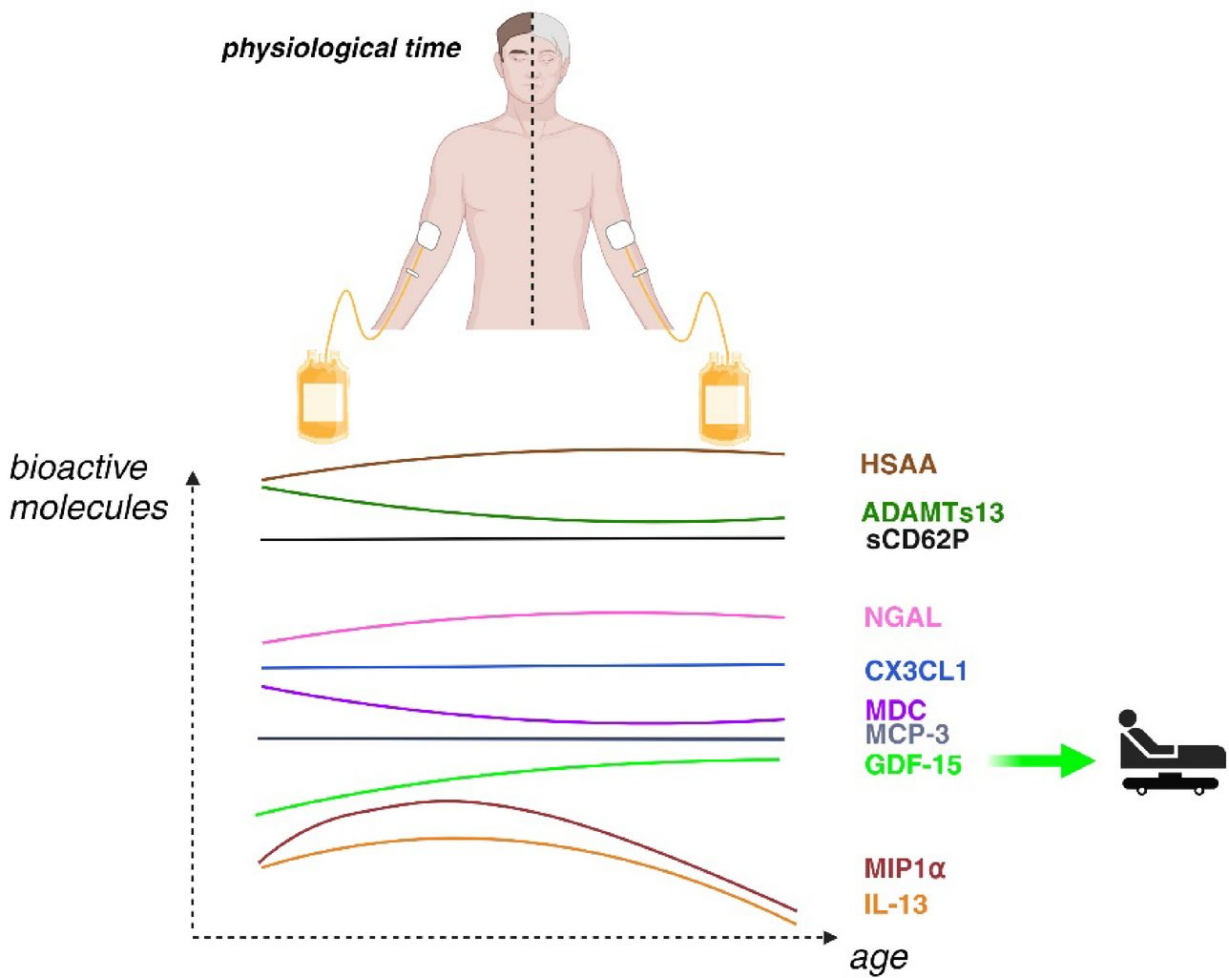
Summary of the study. Our study compared the level of sCD62P mainly released by platelet and HSAA, ADAMTs13, NGAL, CX3 CL1, MDC, MCP-3, GDF-15, MIP1α, IL-13 released by other cell type, detected in Single Donor Apheresis Platelet Concentrate, dedicated for transfusion. These molecules are modulated through the donor’s age, and could play a role in adverse reaction following a transfusion such as GDF-15.

Over the past decade, the aging process has garnered increasing attention and investigation. In animal model, transfusion of younger blood rejuvenate aged mice recipient, for muscular regeneration^18,19^, vascular and neurogenic function^20^, cognitive function^21^and with PF4 level linked with donor’s age^22^. In this study, we focused on the presence of several bioactive molecule released by platelets (sCD62P, NGAL, MIP-1α, and GDF-15) and by leukocytes (MDC, MIP-1α, MCP-3, GDF-15, NGAL, IL-13, CX3 CL1), as well as ADAMTS13, which is primarily released by hepatocytes, endothelial cells, and the megakaryocyte lineage, in SDA-PC from donors of different ages (Fig. 4). The preparation of SDA-PC involved a pathogen inactivation step, which could potentially influence cytokine/chemokine levels. Some studies have shown that the type of platelet additive solution (PAS) used can influence the concentrations of cytokines and chemokines in platelet concentrates. For example, the use of certain additive solutions can reduce levels of pro-inflammatory cytokines, thereby decreasing the risk of adverse transfusion reactions in patients. However, results vary between studies, and it is essential to consider the specific properties of each PAS. The choice of PAS in the preparation of platelet concentrates can have a significant impact on the cytokine and chemokine profile, potentially affecting the recipient’s immune response. Unfortunately, we were unable to collect this information in the present study, which represents one of its limitations. A thorough understanding of these interactions is crucial to optimizing the safety and efficacy of platelet transfusions^23^. Specifically, the PAS-D solution has been shown to decrease sCD62P levels, while increasing sCD40L levels^10^. Furthermore, the storage time of SDA-PC may influence the levels of bioactive molecules, as previously reported^8,9,17^. However, we did not observe significant differences in bioactive molecule modulation based on donor age group or storage duration. Notably, storage time did not affect cytokine levels, with the exception of NGAL and GDF-15^8^. In contrast, our study identified significant modulation of bioactive molecule levels related to donor age, including IL-13, MIP1α, MDC, GDF-15, and sCD62P (Fig. 1A).

Interestingly, we observed that the levels of certain molecules such as ADAMTS13 and MDC present in the PC’s supernatants decrease with donor age (Figs. 1, 2, 3 and 4). Conversely, molecules such as sCD62P, CX3 CL1, and MCP-3 did not show significant modulation with age. On the other hand, levels of HSAA, NGAL, and GDF-15 were found to increase over the lifespan. Interestingly, the age and sex of donors may affect the survival of transfusion recipients with Red Blood Cell or plasma, according to a Canadian study, while a Scandinavian study presents contrasting findings^24–26^. However, for platelet transfusion few publications highlighted the impact of donor sex and age on platelet transfusion^27,28^.

### The most abundant biomolecule (HSAA and ADAMTs13) in our study: implications and interactions

Curiously, HSAA is not produced by platelets themselves, as mRNA or protein for HSAA has not been detected in platelets^29–31^. Its presence in SDA-PC suggests that it originates from plasma, as SDA-PC is comprised of approximately 30% plasma from the donor. Although its levels are not modulated with donor age (Fig. 1A), it remains the most abundant biomolecule in our study (Fig. 1C). Its functions are broad with interaction with fibrinogen^32^, with platelet^33^notably with inhibition of platelet aggregation^34^. Studies in animals and humans have demonstrated that HSAA expression increases with age^35–37^ and is involved in the senescence process.

### Molecules in SDA-PC involved in coagulation and thrombosis processes

In parallel, ADAMTs13 is the second most abundant biomolecule in our study (Fig. 1C), despite its levels not being modulated by the donor’s age (Fig. 1A). However, ADAMTs13 is a cleaving protease specific for von Willebrand factor (vWF), crucial for regulating clot formation. Aging contributes to endothelial dysfunction, affecting the balance between vWF and ADAMTS13^38^. This imbalance is associated with an increased risk of venous and arterial thrombosis^39,40^ underscoring the importance of ADAMTs13 in both aging and transfusion contexts. Finally, and importantly, this dual role of fractalkine in inflammation and thrombosis suggests its potential influence on adverse reactions post-transfusion (Figs. 2 and 3B), and its correlation with platelet activation markers such as sCD62P (Fig. 3A). While sCD62P is extensively studied in the context of platelet transfusion, its role in aging has been less explored. Furthermore, ADAMTs13 and CX3 CL1 have been implicated in cardiovascular complications^41^. Additionally, while an increase in CX3 CL1 levels in elderly donors has been reported in other studies^42^, this was not observed in our study with SDA-PC.

Overall, these findings highlight the significant role of donor age in modulating bioactive molecule levels in SDA-PC and their potential impact on transfusion-related adverse reactions. Understanding the roles of ADAMTS13, CX3 CL1, and sCD62P in SDA-PC is critical for elucidating their contributions to coagulation, thrombosis, and potential adverse reactions in transfusion recipients, particularly in the context of an aging population. Further research is essential to clarify their dynamics in aging and their implications for transfusion medicine and clinical outcomes.

### Molecules contained in SDA-PC involved in immunity and inflammation

It is noteworthy that consistent with other studies IL-13 levels increased in SDA-PC with AR^15,43^and this cytokine is not produced by platelets themselves due to the absence of protein expression despite mRNA presence^29–31^. IL-13 can influence platelet function indirectly by upregulating GPIIb expression on megakaryocytes^44^,. This cytokine inhibits the expression of PECAM1 (platelet endothelial cell adhesion molecule) and increases the permeability of endothelial monolayers^45^. These effects explain its involvement in adverse reactions post-transfusion, as depicted in Fig. 2. Moreover, MIP1α is released by monocytes, dendritic cells, lymphocytes but also platelets, as it is contained within alpha granules and release upon activation^46^. Platelets themselves express receptors for MIP1α^47^, suggesting that this bioactive molecule may modulate their functions. In the context of transfusion, MIP1α could potentially influence platelet activation and interaction with other immune cells, as in AR. Otherwise, MDC is released by monocyte and dendritic cells. It has been shown to induce platelet activation activation^48,49^and regulated Th2 and Treg^50^. In transfusion scenarios, platelet concentrates may modulate myeloid dendritic cell responses through MDC, affecting immune regulation and potentially contributing to clinical outcomes^51^. As for MCP-3 can be released by platelets and monocytes^52^. This bioactive molecule interacts with receptors on both platelets and various leukocytes, playing roles in immune cell recruitment and activation. Like MDC and MIP1α, MCP-3 levels are known to increase in the serum of elderly^53^^54^,,suggesting potential implications in transfusion-related adverse reactions^47^. HSAA has been shown to interact with Toll-like receptors (TLR2 and TLR4) on the surface of various cells including with platelets^55^. Like lipopolysaccharide (LPS), which activates TLR4 to induce the release of sCD40L from platelets^56^ HSAA shares the same receptor characteristics. Therefore, HSAA, as damage-associated molecular pattern (DAMP), might also stimulate the release of sCD40L in SDA-PC through TLR4 activation pathways.

Interestingly GDF-15, play significant roles through their release from different cellular sources and their implications in aging and health. GDF-15 is released by endothelial cells and is associated with conditions such as aging and anemia^57,58^. It is part of the senescence-associated secretory phenotype (SASP) released by senescent cells^59–62^, contributing to senescence induction and associated with longevity^63^. While IL-13 levels decrease with donor age, another study suggests that IL-13 promotes cellular senescence^64^and reports elevated IL-13 levels in the serum of older donors compared to younger ones^65^.

NGAL can be released by platelets and is predominantly produced by monocytes and neutrophils^66,67^. It has been implicated in various physiological processes, including brain aging, where its levels in urine were found to be higher in elderly men compared to elderly women (> 65 years old)^68^. This significant increase of NGAL level in AR compare to no AR could be explained. Moreocer, CX3 CL1 binds to von Willebrand receptor glycoprotein Ib^69^and integrins αvβ3 and αIIbβ3^69^. Fractalkine mediates leukocyte adhesion to endothelium^70^with or without platelets, also involved in platelet activation and adhesion^71^, is potentially inducing vascular dysfunction and releasing superoxide anions.

These findings provide important insights into how the aging process affects the bioactive molecule composition of SDA-PC and suggest that the age of blood donors could influence the quality, yield and safety of transfusion products. This information is crucial for optimizing transfusion practices, particularly in the context of an aging population. Interestingly, the age and sex of donors may affect the survival of transfusion recipients, according to a Canadian study, while a Scandinavian study presents contrasting findings^24–26^. In these studies, red blood cell transfusions were examined. Our manuscript focuses on the impact of donor age and the contents of platelet concentrates on platelet concentrate transfusions, independent of the recipients’ age. Furthermore, in animal model, transfusion of younger blood rejuvenate aged mice recipient, for muscular regeneration^18,19^, vascular and neurogenic function^20^, cognitive function^21^ and so one.

Presence and potential interactions of these bioactive molecules with platelets and other immune cells highlight their significance in transfusion medicine. Understanding their roles in the context of aging and their impact on immune responses following transfusion is essential for optimizing transfusion practices and improving patient outcomes.

## Electronic supplementary material

Below is the link to the electronic supplementary material.

Supplementary Material 1

## Data Availability

Any additional information required to reanalyse the data reported in this work paper is available from the lead contact (corresponding authors) upon request.

## References

[1] Kleymenov, D. A. et al. A deep look into COVID-19 severity through dynamic changes in blood cytokine levels. *Front. Immunol.* **12**, 771609. 10.3389/fimmu.2021.771609 (2021). 34858428 10.3389/fimmu.2021.771609 PMC8630739

[2] Huang, C. et al. Multi-cohort study on cytokine and chemokine profiles in the progression of COVID-19. *Sci. Rep.* **14**, 10324. 10.1038/s41598-024-61133-z (2024). 38710800 10.1038/s41598-024-61133-z PMC11074324

[3] Cisneros, B. et al. Immune system modulation in aging: molecular mechanisms and therapeutic targets. *Front. Immunol.* **13**, 1059173. 10.3389/fimmu.2022.1059173 (2022). 36591275 10.3389/fimmu.2022.1059173 PMC9797513

[4] Mogilenko, D. A., Shchukina, I. & Artyomov, M. N. Immune ageing at single-cell resolution. *Nat. Rev. Immunol.* **22**, 484–498. 10.1038/s41577-021-00646-4 (2022). 34815556 10.1038/s41577-021-00646-4 PMC8609266

[5] Li, X. et al. Inflammation and aging: signaling pathways and intervention therapies. *Signal. Transduct. Target. Ther.* **8**, 239. 10.1038/s41392-023-01502-8 (2023). 37291105 10.1038/s41392-023-01502-8 PMC10248351

[6] World-Health-Organization. *Blood safety and availability - Blood transfusion*, (2023). https://www.who.int/news-room/fact-sheets/detail/blood-safety-and-availability#:~:text=Blood%20transfusions,-There%20are%20great&text=For%20example%2C%20in%20high%2Dincome,the%20age%20of%205%20years.&gt

[7] World-Health-Organization. *Global status report on blood safety and availability 2021*, (2022). https://www.who.int/publications/i/item/9789240051683

[8] Duchez, A. C. et al. Identification of new bioactive molecules in platelet preparation, storage, and transfusion reactions for improved transfusion management. *Sci. Rep.* **14**, 29381. 10.1038/s41598-024-80632-7 (2024). 39592728 10.1038/s41598-024-80632-7 PMC11599570

[9] Duchez, A. C. et al. Bioactive lipids as biomarkers of adverse reactions associated with apheresis platelet concentrate transfusion. *Front. Immunol.* **14**, 1031968. 10.3389/fimmu.2023.1031968 (2023). 37138863 10.3389/fimmu.2023.1031968 PMC10149858

[10] Sut, C. et al. Soluble CD40L and CD62P levels differ in single-donor apheresis platelet concentrates and Buffy coat-derived pooled platelet concentrates. *Transfusion* **59**, 16–20. 10.1111/trf.14974 (2019). 30291753 10.1111/trf.14974

[11] Xu, J. et al. [Cytokine contents in single donor platelets during storage]. *Zhongguo Shi Yan Xue Ye Xue Za Zhi*. **16**, 1185–1187 (2008). 18928624

[12] Fujihara, M., Ikebuchi, K., Wakamoto, S. & Sekiguchi, S. Effects of filtration and gamma radiation on the accumulation of RANTES and transforming growth factor-beta1 in apheresis platelet concentrates during storage. *Transfusion* **39**, 498–505. 10.1046/j.1537-2995.1999.39050498.x (1999). 10336000 10.1046/j.1537-2995.1999.39050498.x

[13] Wadhwa, M. et al. Cytokine levels in platelet concentrates: quantitation by bioassays and immunoassays. *Br. J. Haematol.* **93**, 225–234. 10.1046/j.1365-2141.1996.4611002.x (1996). 8611466 10.1046/j.1365-2141.1996.4611002.x

[14] Cognasse, F. et al. Release of potential Immunomodulatory factors during platelet storage. *Transfusion* **46**, 1184–1189. 10.1111/j.1537-2995.2006.00869.x (2006). 16836566 10.1111/j.1537-2995.2006.00869.x

[15] Nguyen, K. A. et al. A computerized prediction model of hazardous inflammatory platelet transfusion outcomes. *PLoS One*. **9**, e97082. 10.1371/journal.pone.0097082 (2014). 24830754 10.1371/journal.pone.0097082 PMC4022636

[16] Cognasse, F. et al. Platelet soluble CD40-ligand level is associated with transfusion adverse reactions in a mixed threshold-and-hit model. *Blood* **130**, 1380–1383. 10.1182/blood-2017-03-773945 (2017). 28720587 10.1182/blood-2017-03-773945

[17] Duchez, A. C. et al. Lipidomic analysis of differently prepared platelet concentrates in additive solution during storage. *Blood Transfus.*10.2450/2022.0144-22 (2022). 36346879 10.2450/2022.0144-22 PMC10497391

[18] Conboy, I. M. et al. Rejuvenation of aged progenitor cells by exposure to a young systemic environment. *Nature* **433**, 760–764. 10.1038/nature03260 (2005). 15716955 10.1038/nature03260

[19] Sinha, M. et al. Restoring systemic GDF11 levels reverses age-related dysfunction in mouse skeletal muscle. *Science* **344**, 649–652. 10.1126/science.1251152 (2014). 24797481 10.1126/science.1251152 PMC4104429

[20] Katsimpardi, L. et al. Vascular and neurogenic rejuvenation of the aging mouse brain by young systemic factors. *Science* **344**, 630–634. 10.1126/science.1251141 (2014). 24797482 10.1126/science.1251141 PMC4123747

[21] Leiter, O. et al. Platelet-derived exerkine CXCL4/platelet factor 4 rejuvenates hippocampal neurogenesis and restores cognitive function in aged mice. *Nat. Commun.* **14**, 4375. 10.1038/s41467-023-39873-9 (2023). 37587147 10.1038/s41467-023-39873-9 PMC10432533

[22] Duchez, A. C. et al. In platelet single donor apheresis, platelet factor 4 levels correlated with donor’s age and decreased during storage. *Sci. Rep.* **14**, 6231. 10.1038/s41598-024-56826-4 (2024). 38485973 10.1038/s41598-024-56826-4 PMC10940288

[23] Garraud, O. et al. Platelet transfusion in adults: an update. *Transfus. Clin. Biol.* **30**, 147–165. 10.1016/j.tracli.2022.08.147 (2023). 36031180 10.1016/j.tracli.2022.08.147

[24] Edgren, G. et al. Association of donor age and sex with survival of patients receiving transfusions. *JAMA Intern. Med.* **177**, 854–860. 10.1001/jamainternmed.2017.0890 (2017). 28437543 10.1001/jamainternmed.2017.0890 PMC5540056

[25] Vasan, S. K. et al. Lack of association between blood donor age and survival of transfused patients. *Blood* **127**, 658–661. 10.1182/blood-2015-11-683862 (2016). 26702060 10.1182/blood-2015-11-683862

[26] Chasse, M. et al. Association of blood donor age and sex with recipient survival after red blood cell transfusion. *JAMA Intern. Med.* **176**, 1307–1314. 10.1001/jamainternmed.2016.3324 (2016). 27398639 10.1001/jamainternmed.2016.3324

[27] D’Alessandro, A., Stefanoni, D., Slichter, S. J., Fu, X. & Zimring, J. C. The impact of donor sex and age on stored platelet metabolism and post-transfusion recovery. *Blood Transfus.* **19**, 216–223. 10.2450/2020.0145-20 (2021). 33085601 10.2450/2020.0145-20 PMC8092042

[28] Bontekoe, I. J., van der Meer, P. F., Verhoeven, A. J. & de Korte, D. Platelet storage properties are associated with donor age: in vitro quality of platelets from young donors and older donors with and without type 2 diabetes. *Vox Sang*. **114**, 129–136. 10.1111/vox.12739 (2019). 30536565 10.1111/vox.12739

[29] Supernat, A. et al. Transcriptomic landscape of blood platelets in healthy donors. *Sci. Rep.* **11**, 15679. 10.1038/s41598-021-94003-z (2021). 34344933 10.1038/s41598-021-94003-z PMC8333095

[30] Huang, J. et al. Assessment of a complete and classified platelet proteome from genome-wide transcripts of human platelets and megakaryocytes covering platelet functions. *Sci. Rep.* **11**, 12358. 10.1038/s41598-021-91661-x (2021). 34117303 10.1038/s41598-021-91661-x PMC8196183

[31] Rowley, J. W. et al. Genome-wide RNA-seq analysis of human and mouse platelet transcriptomes. *Blood* **118**, e101–111. 10.1182/blood-2011-03-339705 (2011). 21596849 10.1182/blood-2011-03-339705 PMC3193274

[32] Page, M. J. et al. Serum amyloid A binds to fibrin(ogen), promoting fibrin amyloid formation. *Sci. Rep.* **9**, 3102. 10.1038/s41598-019-39056-x (2019). 30816210 10.1038/s41598-019-39056-x PMC6395759

[33] Urieli-Shoval, S. et al. Adhesion of human platelets to serum amyloid A. *Blood* **99**, 1224–1229. 10.1182/blood.v99.4.1224 (2002). 11830469 10.1182/blood.v99.4.1224

[34] Sayinalp, N. et al. Protein C inhibitor and serum amyloid A in immune thrombocytopaenic purpura. *J. Int. Med. Res.* **32**, 62–65. 10.1177/147323000403200110 (2004). 14997708 10.1177/147323000403200110

[35] Asahi, Y., Arai, T. & Tanaka, Y. Changes in plasma metabolite concentrations and enzyme activities in aging riding horses. *Front. Vet. Sci.* **11**, 1345548. 10.3389/fvets.2024.1345548 (2024). 38881783 10.3389/fvets.2024.1345548 PMC11177609

[36] Hogarth, M. B. et al. Acute phase proteins, C-reactive protein and serum amyloid A protein, as prognostic markers in the elderly inpatient. *Age Ageing*. **26**, 153–158. 10.1093/ageing/26.2.153 (1997). 9177673 10.1093/ageing/26.2.153

[37] Hijmans, W. & Sipe, J. D. Levels of the serum amyloid A protein (SAA) in normal persons of different age groups. *Clin. Exp. Immunol.* **35**, 96–100 (1979). 428149 PMC1537586

[38] Thangaraju, K. et al. The impact of age and BMI on the VWF/ADAMTS13 axis and simultaneous thrombin and plasmin generation in hospitalized COVID-19 patients. *Front. Med. (Lausanne)*. **8**, 817305. 10.3389/fmed.2021.817305 (2021). 35087853 10.3389/fmed.2021.817305 PMC8786628

[39] Calabro, P., Gragnano, F., Golia, E. & Grove, E. L. Von Willebrand factor and venous thromboembolism: pathogenic link and therapeutic implications. *Semin Thromb. Hemost.* **44**, 249–260. 10.1055/s-0037-1605564 (2018). 28898897 10.1055/s-0037-1605564

[40] Ni, X. et al. Identification and replication of novel genetic variants of ABO gene to reduce the incidence of diseases and promote longevity by modulating lipid homeostasis. *Aging (Albany NY)*. **13**, 24655–24674. 10.18632/aging.203700 (2021). 34812738 10.18632/aging.203700 PMC8660604

[41] Rawish, E., Nording, H., Munte, T. & Langer, H. F. Platelets as mediators of neuroinflammation and thrombosis. *Front. Immunol.* **11**, 548631. 10.3389/fimmu.2020.548631 (2020). 33123127 10.3389/fimmu.2020.548631 PMC7572851

[42] Chen, X. et al. CX3C chemokine: hallmarks of fibrosis and ageing. *Pharmacol. Res.* **208**, 107348. 10.1016/j.phrs.2024.107348 (2024). 39134186 10.1016/j.phrs.2024.107348

[43] Cognasse, F. et al. Platelet components associated with adverse reactions: predictive value of mitochondrial DNA relative to biological response modifiers. *Transfusion* **56**, 497–504. 10.1111/trf.13373 (2016). 26446055 10.1111/trf.13373

[44] Almas, S. et al. Immunofluorescence analysis of human eosinophils. *J. Immunol. Methods*. **526**, 113619. 10.1016/j.jim.2024.113619 (2024). 38272178 10.1016/j.jim.2024.113619

[45] Ren, Q. et al. Platelet endothelial cell adhesion molecule-1 (PECAM1) plays a critical role in the maintenance of human vascular endothelial barrier function. *Cell. Biochem. Funct.* **33**, 560–565. 10.1002/cbf.3155 (2015). 26607202 10.1002/cbf.3155

[46] Klinger, M. H. et al. Immunocytochemical localization of the chemokines RANTES and MIP-1 alpha within human platelets and their release during storage. *Int. Arch. Allergy Immunol.* **107**, 541–546. 10.1159/000237097 (1995). 7542516 10.1159/000237097

[47] Boehlen, F. & Clemetson, K. J. Platelet chemokines and their receptors: what is their relevance to platelet storage and transfusion practice? *Transfus. Med.* **11**, 403–417. 10.1046/j.1365-3148.2001.00340.x (2001). 11851938 10.1046/j.1365-3148.2001.00340.x

[48] Abi-Younes, S., Si-Tahar, M. & Luster, A. D. The CC chemokines MDC and TARC induce platelet activation via CCR4. *Thromb. Res.* **101**, 279–289. 10.1016/s0049-3848(00)00402-3 (2001). 11248289 10.1016/s0049-3848(00)00402-3

[49] Rosa, A. et al. WASp controls oriented migration of endothelial cells to achieve functional vascular patterning. *Development* **149**10.1242/dev.200195 (2022). 10.1242/dev.200195 PMC8918813 34931661

[50] Li, Q. et al. Regulation of Th1/Th2 and Th17/Treg by pDC/mDC imbalance in primary immune thrombocytopenia. *Exp. Biol. Med. (Maywood)*. **246**, 1688–1697. 10.1177/15353702211009787 (2021). 33938255 10.1177/15353702211009787 PMC8719040

[51] Ki, K. K., Faddy, H. M., Flower, R. L. & Dean, M. M. Platelet concentrates modulate myeloid dendritic cell immune responses. *Platelets* **29**, 373–382. 10.1080/09537104.2017.1306045 (2018). 28503991 10.1080/09537104.2017.1306045

[52] Gear, A. R. & Camerini, D. Platelet chemokines and chemokine receptors: linking hemostasis, inflammation, and host defense. *Microcirculation* **10**, 335–350. 10.1038/sj.mn.7800198 (2003). 12851650 10.1038/sj.mn.7800198

[53] Chulenbayeva, L. et al. The trajectory of successful aging: insights from metagenome and cytokine profiling. *Gerontology* **70**, 390–407. 10.1159/000536082 (2024). 38246133 10.1159/000536082 PMC11008724

[54] Coperchini, F. et al. Inflamm-ageing: how cytokines and nutrition shape the trajectory of ageing. *Cytokine Growth Factor. Rev.*10.1016/j.cytogfr.2024.08.004 (2024). 39237438 10.1016/j.cytogfr.2024.08.004

[55] Cognasse, F. et al. Evidence of Toll-like receptor molecules on human platelets. *Immunol. Cell. Biol.* **83**, 196–198. 10.1111/j.1440-1711.2005.01314.x (2005). 15748217 10.1111/j.1440-1711.2005.01314.x

[56] Cognasse, F., Lafarge, S., Chavarin, P., Acquart, S. & Garraud, O. Lipopolysaccharide induces sCD40L release through human platelets TLR4, but not TLR2 and TLR9. *Intensive Care Med.* **33**, 382–384. 10.1007/s00134-006-0488-8 (2007). 17180393 10.1007/s00134-006-0488-8

[57] Fukuda, T. et al. Physiological role of serum growth differentiation Factor-15 (GDF-15) level and iron metabolism in Community-Dwelling older adults. *Cureus* **16**, e60422. 10.7759/cureus.60422 (2024). 38883134 10.7759/cureus.60422 PMC11179486

[58] Wang, L. et al. Circulating GDF-15: a biomarker for metabolic dysregulation and aging in people living with HIV. *Front. Aging*. **5**, 1414866. 10.3389/fragi.2024.1414866 (2024). 38895099 10.3389/fragi.2024.1414866 PMC11183798

[59] Sarad, K. et al. Senescence of endothelial cells promotes phenotypic changes in adventitial fibroblasts: possible implications for vascular aging. *Mol. Cell. Biochem.*10.1007/s11010-024-05028-7 (2024). 38743322 10.1007/s11010-024-05028-7 PMC11835997

[60] Chiariello, A. et al. Downregulation of PLIN2 in human dermal fibroblasts impairs mitochondrial function in an age-dependent fashion and induces cell senescence via GDF15. *Aging Cell.* **23**, e14111. 10.1111/acel.14111 (2024). 38650174 10.1111/acel.14111 PMC11113257

[61] Evans, D. S. et al. Proteomic analysis of the Senescence-Associated secretory phenotype: GDF-15, IGFBP-2, and Cystatin-C are associated with multiple aging traits. *J. Gerontol. Biol. Sci. Med. Sci.* **79**10.1093/gerona/glad265 (2024). 10.1093/gerona/glad265 PMC10876076 37982669

[62] Nyarady, B. B. et al. Growth and differentiation factor-15: A link between inflammaging and cardiovascular disease. *Biomed. Pharmacother*. **174**, 116475. 10.1016/j.biopha.2024.116475 (2024). 38522236 10.1016/j.biopha.2024.116475

[63] Liu, X. et al. Plasma proteomic signature of human longevity. *Aging Cell.* **23**, e14136. 10.1111/acel.14136 (2024). 38440820 10.1111/acel.14136 PMC11166369

[64] Zhu, M. et al. Interleukin-13 promotes cellular senescence through inducing mitochondrial dysfunction in IgG4-related sialadenitis. *Int. J. Oral Sci.* **14**, 29. 10.1038/s41368-022-00180-6 (2022). 35718799 10.1038/s41368-022-00180-6 PMC9207030

[65] Li, Q., Liu, X. & Wei, J. Ageing related Periostin expression increase from cardiac fibroblasts promotes cardiomyocytes senescent. *Biochem. Biophys. Res. Commun.* **452**, 497–502. 10.1016/j.bbrc.2014.08.109 (2014). 25173938 10.1016/j.bbrc.2014.08.109

[66] Dekens, D. W. et al. Lipocalin 2 as a link between ageing, risk factor conditions and age-related brain diseases. *Ageing Res. Rev.* **70**, 101414. 10.1016/j.arr.2021.101414 (2021). 34325073 10.1016/j.arr.2021.101414

[67] Chen, M. et al. Plasma level of Lipocalin 2 is increased in neovascular age-related macular degeneration patients, particularly those with macular fibrosis. *Immun. Ageing*. **17**, 35. 10.1186/s12979-020-00205-w (2020). 33292361 10.1186/s12979-020-00205-w PMC7666483

[68] Czarkowska-Paczek, B., Wyczalkowska-Tomasik, A. & Paczek, L. Laboratory blood test results beyond normal ranges could not be attributed to healthy aging. *Med. (Baltim).* **97**, e11414. 10.1097/MD.0000000000011414 (2018). 10.1097/MD.0000000000011414 PMC6076198 29995788

[69] dos Meyer, S. et al. The CX3C chemokine fractalkine mediates platelet adhesion via the von Willebrand receptor glycoprotein Ib. *Blood* **117**, 4999–5008. 10.1182/blood-2011-02-335471 (2011). 21398580 10.1182/blood-2011-02-335471

[70] Schulz, C. et al. Chemokine fractalkine mediates leukocyte recruitment to inflammatory endothelial cells in flowing whole blood: a critical role for P-selectin expressed on activated platelets. *Circulation* **116**, 764–773. 10.1161/CIRCULATIONAHA.107.695189 (2007). 17679613 10.1161/CIRCULATIONAHA.107.695189

[71] Schafer, A. et al. Novel role of the membrane-bound chemokine fractalkine in platelet activation and adhesion. *Blood* **103**, 407–412. 10.1182/blood-2002-10-3260 (2004). 12969973 10.1182/blood-2002-10-3260

