# Supplementary material for: The composition of single-donor apheresis platelet concentrates is influenced by the age of the donor
Source: Sci Rep. 2025 Apr 18;15:13505. doi: 10.1038/s41598-025-97916-1 (PMC12008385; doi:10.1038/s41598-025-97916-1)
Supplement: Supplementary file 1 — Supplementary Material 1 [file 41598_2025_97916_MOESM1_ESM.docx]

**
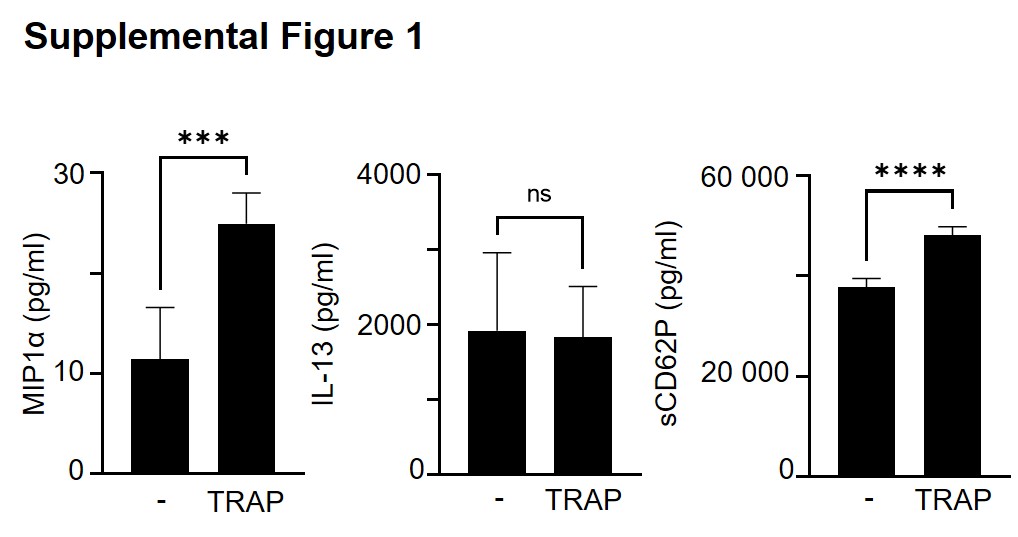
**

**Supplemental Figure 1. Under stimulation, platelets released BRMs.**

Platelet rich plasma stimulated with TRAP released several molecules. *** p<0.001 ****p<0.0001 Wilcoxon paired test. *n=*11/17 for MIP1α analysis ; *n=*4 for IL-13 ananlysis; *n=*17 for sCD62P analysis


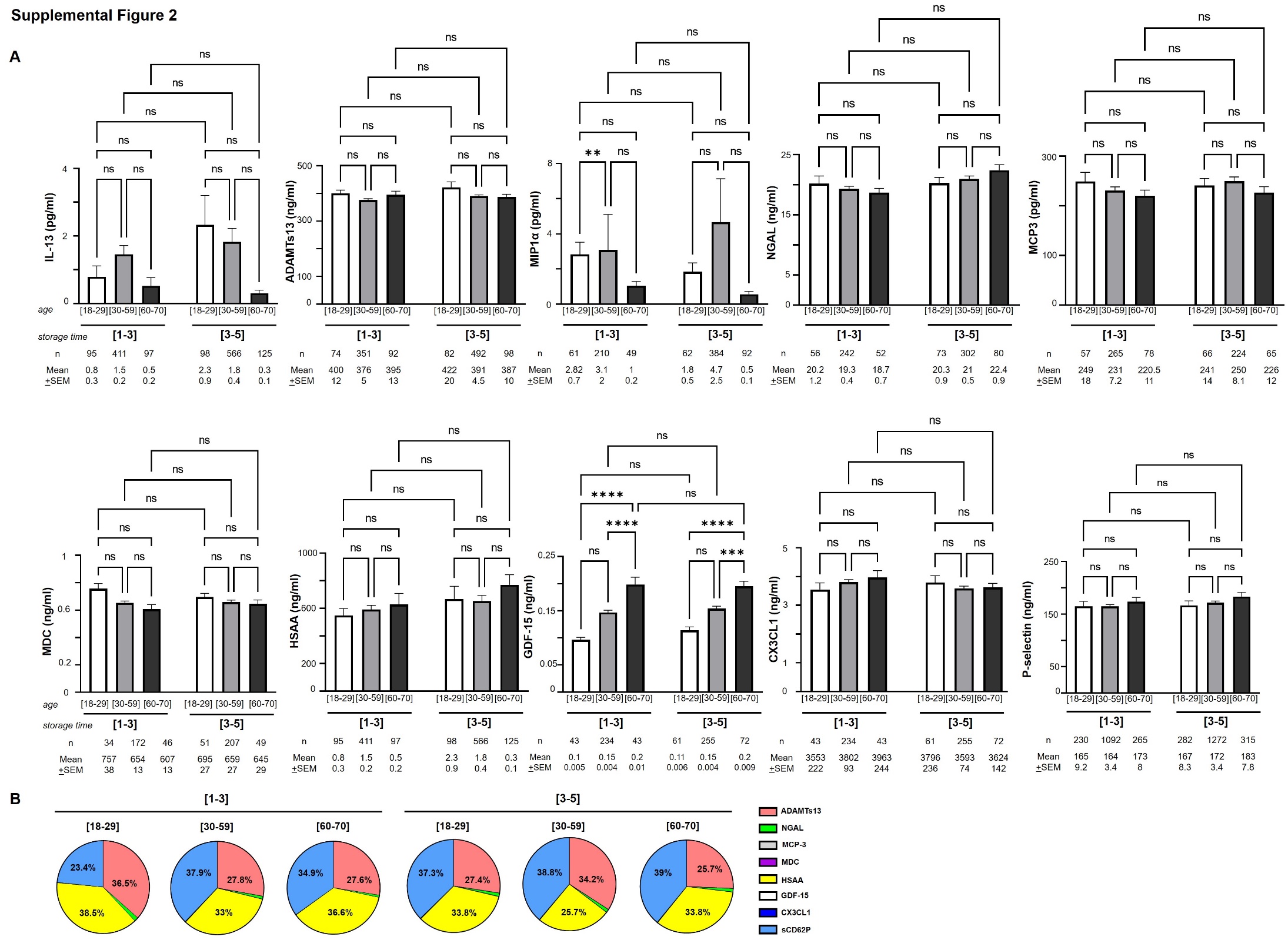


**Supplemental Figure 2. Evaluation of cytokines in Single Donor Apheresis based on donor’s age and storage time.** (A) Graph bars representing the concentration of cytokine in SDA-PC along the donor’s age and storage time. 2-way ANOVA test with Tukey, **p<0.01; ***p<0.001; ****p<0,0001. (B) Pie chart representing the distribution of the cytokines evaluated in SDA-PC with donor’s age and storage time.


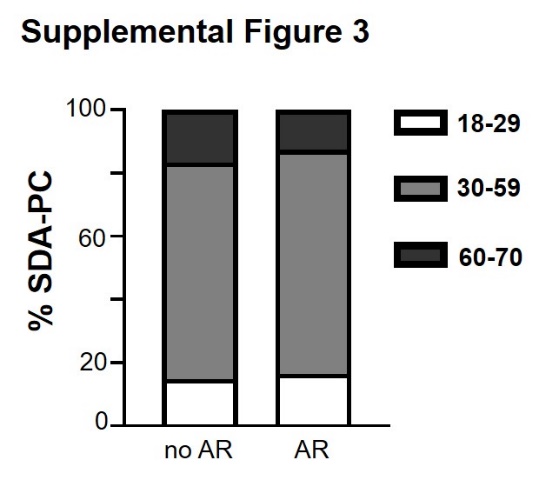


**Supplemental Figure 3. Evaluation of age donor’s impact on Adverse Reaction following a SDA-PC transfusion.** Graph bars representing the pourcentage of SDA-PC while AR concern and donor’s age.
